# Cell Tracking in Cancer Immunotherapy

**DOI:** 10.3389/fmed.2020.00034

**Published:** 2020-02-14

**Authors:** Justine Perrin, Marisa Capitao, Marie Mougin-Degraef, François Guérard, Alain Faivre-Chauvet, Latifa Rbah-Vidal, Joëlle Gaschet, Yannick Guilloux, Françoise Kraeber-Bodéré, Michel Chérel, Jacques Barbet

**Affiliations:** ^1^CRCINA, INSERM, CNRS, Université d'Angers, Université de Nantes, Nantes, France; ^2^Nuclear Medicine, University Hospital, Nantes, France; ^3^Nuclear Medicine, ICO Cancer Center, Saint-Herblain, France; ^4^GIP Arronax, Saint-Herblain, France

**Keywords:** cell tracking, immunotherapy, PET, SPECT, MRI, adoptive transfer, tumor microenvironment, cancer

## Abstract

The impressive development of cancer immunotherapy in the last few years originates from a more precise understanding of control mechanisms in the immune system leading to the discovery of new targets and new therapeutic tools. Since different stages of disease progression elicit different local and systemic inflammatory responses, the ability to longitudinally interrogate the migration and expansion of immune cells throughout the whole body will greatly facilitate disease characterization and guide selection of appropriate treatment regiments. While using radiolabeled white blood cells to detect inflammatory lesions has been a classical nuclear medicine technique for years, new non-invasive methods for monitoring the distribution and migration of biologically active cells in living organisms have emerged. They are designed to improve detection sensitivity and allow for a better preservation of cell activity and integrity. These methods include the monitoring of therapeutic cells but also of all cells related to a specific disease or therapeutic approach. Labeling of therapeutic cells for imaging may be performed *in vitro*, with some limitations on sensitivity and duration of observation. Alternatively, *in vivo* cell tracking may be performed by genetically engineering cells or mice so that may be revealed through imaging. In addition, SPECT or PET imaging based on monoclonal antibodies has been used to detect tumors in the human body for years. They may be used to detect and quantify the presence of specific cells within cancer lesions. These methods have been the object of several recent reviews that have concentrated on technical aspects, stressing the differences between direct and indirect labeling. They are briefly described here by distinguishing *ex vivo* (labeling cells with paramagnetic, radioactive, or fluorescent tracers) and *in vivo* (*in vivo* capture of injected radioactive, fluorescent or luminescent tracers, or by using labeled antibodies, ligands, or pre-targeted clickable substrates) imaging methods. This review focuses on cell tracking in specific therapeutic applications, namely cell therapy, and particularly CAR (Chimeric Antigen Receptor) T-cell therapy, which is a fast-growing research field with various therapeutic indications. The potential impact of imaging on the progress of these new therapeutic modalities is discussed.

## Introduction

The origins of immunotherapy go back to early centuries of history as illustrated by the fight against smallpox. Realization that survivors were immune to the disease eventually led to the practice of inoculation or variolation, that spread throughout Europe in the early eighteenth century. The discovery of cowpox vaccination by Edward Jenner in 1796 ultimately resulted, after a global vaccination campaign, in the eradication of the disease announced by the World Health Organization in 1977. Fighting infectious diseases with vaccines proved successful, but eradication of other diseases remains elusive. While Jonas Salk developed the first poliomyelitis vaccine in the 1950, the disease is not yet considered as eradicated and remains endemic in several African countries (1). In the meantime, the role of immunity in other pathologies has been explored and the immune system is now identified as a general defense system that distinguishes self from non-self or altered self. Its ability to recognize normal cells from infected or tumor cells has implications in cancer immune surveillance, graft rejection, and many other pathologies but can also result in autoimmune, and inflammatory diseases. It was also realized that the immune system uses an incredibly complex network of connected cellular and molecular agents, not yet fully known and understood.

The focus of this review is on anti-cancer immunotherapy as it is making impressive progress. However, the concepts can also be paralleled in other immune-mediated disorders and for conditions requiring immunotherapeutic intervention. Therapeutic antibodies and cell-based therapies, such as adoptive immunotherapy and stem-cell therapy, have been developed years ago, but, in the last few years, a more precise understanding of control mechanisms of the immune system triggered an impressive development of immunotherapy (2). Novel therapeutic approaches have recently emerged that reached clinical practice with remarkable success in a variety of cancers (3, 4). The different types of tissue injuries and the different stages of disease progression are more precisely identified, as well as the different local and systemic inflammatory responses. Monitoring the depletion, migration, and expansion of immune cells throughout the whole body should help characterizing the diseases and guiding selection of appropriate treatment regiments (5). Such methods have an important role in basic cancer research, where they serve to elucidate novel biological mechanisms. The development of effective therapeutic strategies, targeting tumor cells as well as their micro-environment, also requires the ability to determine *in vivo* the location, distribution, and long-term viability of the cell populations as well as their biological fate with respect to cell activation and differentiation.

This process is referred to as cell tracking and is not limited to therapeutic cells but includes all cells related to a specific disease or therapeutic approach, like tumor cells, immune cells or microenvironment. It involves non-invasive methods for monitoring the distribution and migration of biologically active cells in living organisms. In conjunction with various non-invasive imaging modalities, cell-labeling methods, such as exogenous labeling or transfection with a reporter gene, allow visualization of labeled cells *in vivo* in real time, as well as monitoring and quantifying cell accumulation and function by a variety of imaging approaches. In this Review, we briefly describe the basic principles of cell-tracking methods and explain various approaches to cell tracking. Then we highlight recent examples of application of new technologies in animals, focusing on immune checkpoint inhibitor antibodies and cell-based therapies that use natural or genetically engineered T cells, dendritic cells, macrophages or stem cells, and when documented, the clinical potential of these methods.

## Cell Tracking Methods: Looking For Cells in Animal or Human Bodies

Most earlier reviews on this topic have classified imaging techniques as direct or indirect labeling methods. The distinction between direct and indirect labeling is not entirely clear and here we will discuss *ex vivo* vs. *in vivo* labeling: *ex vivo* labeling include labeling cells with paramagnetic, radioactive or fluorescent tracers before injection, while *in vivo* labeling relates to *in situ* imaging cells by injecting radioactive, fluorescent, or luminescent tracers, or antibodies.

SPECT and PET imaging with labeled monoclonal antibodies has been used for years to detect cancer cells. With the development of immuno-PET, they are now used to detect, quantify and longitudinally monitor *in vivo* a variety of cells in the context of immunotherapy of cancer and other diseases (6). Using radiolabeled tracers for *in vivo* imaging will thus be discussed in this review as one of the possible methods of cell tracking.

**Figure 1:**
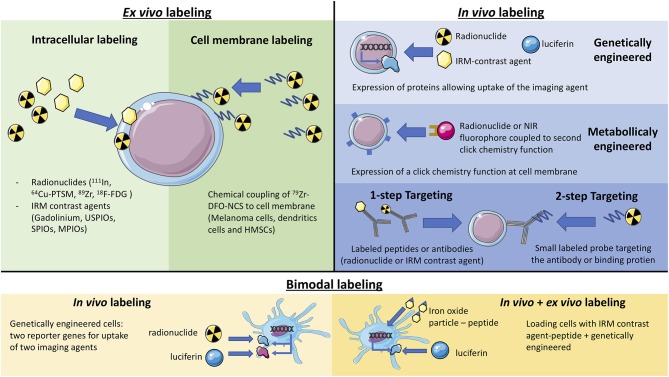
Schematic representation of the different labeling methods (*ex vivo* labeling, *in vivo* labeling, and bimodal).

The various labeling techniques discussed in this review are presented schematically in Figure 1.

### *Ex vivo* Cell Labeling

While the administration of radiolabeled white blood cells has been a classical nuclear medicine technique for years to detect inflammatory lesions (7), new non-invasive methods for monitoring the distribution and migration of biologically active cells in living organisms have emerged. They aim at improving the detection sensitivity and allowing for a better preservation of cell activity and integrity. These methods have been the subject of many reviews (8). Labeling therapeutic cells for imaging may now be performed *in vitro* with little impact on cell function nor migration ability, with some limitations on sensitivity and duration of observation (7, 9, 10). Methods based on radioactive imaging or MRI have the highest potential for clinical imaging. They are briefly presented here in this order, highlighting recent progress.

#### Radioactive (SPECT, PET)

Labeling cells with long-lived radionuclides before re-injection has been used for years in nuclear medicine routine, as mentioned above, but concerns about cell viability and maintenance of cell functions arose. Typically, ^111^In-oxine is used to label leukocytes (11). Cell labeling yield is good, but a significant efflux rate was reported, and image quality is considered suboptimal with this high energy single photon emitter.

Most recent developments relate to cell labeling using positron emitters because, in human, PET imaging offers better resolution and more precise quantification compared to SPECT. Copper-64 is an interesting candidate, with good imaging properties and a relatively long half-life of 12.7 h. ^64^Cu-pyruvaldehyde-bis(N4-methylthiosemicarbazone (^64^Cu-PTSM) was thus used to label C6 glioma cells, as the lipophilic complex is readily taken up in cells. A good cell labeling yield, but a significant efflux rate from cells was observed (12). Zirconium-89 has a half-life of 78.4 h, which is quite convenient to monitor cell trafficking over a few days after administration. Myeloma cells were labeled with ^89^Zr-oxine using a technique similar to that used for In-111 cell labeling (9). Cell labeling yield was reasonable but contrasting results for efflux rate and cell viability were reported. Sato et al. (10) reported that ^89^Zr-oxine complex readily labeled dendritic cells (DC) with an efficiency range of 13.0–43.9 and 83.5% ± 1.8 retention 5 days after labeling. In this study, it was considered that labeling did not affect the viability of mouse DCs and Cytotoxic T Lymphocytes (CTLs), nor did it affect functionality. More recently ^89^Zr-labeled CAR (Chimeric Antigen Receptor) T cells were shown to retain more than 60% of the ^89^Zr over 6 days while their capacity of *in vitro* cytokine production, migration, and tumor cytotoxicity, as well as their *in vivo* antitumor activity (13) were preserved. To further reduce efflux rate and improve viability and cell functions, labeling mixed lymphocyte cell populations with Zr-89 radiolabeled nanoparticles was explored (14, 15).

An alternative approach to loading the radionuclide inside the cells has been proposed. It uses Zr-89-desferrioxamine-NCS, which chemically couples to the membrane of cells. Mouse melanoma cells, dendritic cells and human mesenchymal stem cells were labeled by this method, which was shown to afford stable labeling for 7 days, with little effect of on cell viability and proliferation and to allow for serial PET scans in mouse models (16).

With its fast and efficient uptake and good retention, ^18^F-labeled fluoro-2-deoxy-2-D-glucose (^18^F-FDG) may be used to label cells *in vitro* to monitor cell traffic *in vivo*. For instance, cardiac stem cells were labeled and their biodistribution and retention was quantified in a pig model of chronic myocardial infarction (17). A potential drawback of ^18^F-FDG for assessing cell therapies following implantation is the local retention of radiotracer released from the cells. Thus, 3′-deoxy-3′-L-[^18^F]-fluorothymidine (^18^F-FLT) has been proposed to label cells instead of ^18^F-FDG. Human Umbilical Endothelial Vein Cells (HUVECs) incubated with ^18^F-FLT and injected in mice with hind-limb ischemia were shown to provide a better estimation of HUVECs retention than cells labeled with ^18^F-FDG (18).

#### Magnetic Resonance Imaging (MRI)

Gadolinium(III) chelates, such as gadopentetate dimeglumine, are effective paramagnetic contrast agents owing to their unpaired electrons. These electrons confer a magnetic moment that increases the relaxivity of water protons, shortens the longitudinal relaxation rate (T1) and, therefore, increases the signal by creating a positive contrast in T1-weighted MRI images (19). The amount of gadolinium that may be loaded into cells obviously limits the sensitivity. As an example, rat mesenchymal stem cells (MSC) were loaded *in vitro* with Gd-DTPA using the lipidic transfection agent Effectene. Electron microscopy detected the presence of Gd-DTPA particles in the MSCs and no difference was observed in cell viability or proliferation between the labeled and unlabeled MSCs. T1-weighted MRI was then used to detect the labeled cells *in vitro* and in the rat brain (20).

Superparamagnetic iron-oxide particles have an inherently larger effect on MRI relaxivity than soluble paramagnetic agents. Their core may contain several thousand iron atoms, which increases the local iron concentration and sensitivity. These particles may be coated with dextran, siloxan, citrate, or polymers to improve biodistribution. The superparamagnetic agent results in negative contrast in T2-weighted sequences by causing inhomogeneities in the local magnetic field and spin–spin dephasing, which shortens transverse relaxation times (21). Ultra-small superparamagnetic iron oxide (USPIO) of 10–50 nm, superparamagnetic iron oxide (SPIO) of 50–100 nm and micrometer-sized iron oxide (MPIOs) up to >1 μm particles have been used (8). Again, cell viability limits the intracellular particle concentrations and thus cell detection sensitivity. Phagocytic cells, such as dendritic cells or pancreatic islet cells, can accumulate large amounts of nanoparticles to allow for their detection in animals and patients (22). Macrophages were easily and efficiently labeled with micrometer-sized particles of iron-oxide (MPIO) *in situ* and analyzed via *ex vivo* magnetic resonance microscopy (MRM) and *in vivo* monitoring by magnetic resonance imaging (MRI). The results were confirmed by fluorescence with an anti-macrophage phenotype marker F4/80 antibody (23). Technological improvements in the sensitivity of MRI equipment afforded promising results in detecting smaller numbers of cells that are difficult to label, including T lymphocytes (24).

Chemical exchange saturation transfer has been proposed as a new mechanism for contrast enhancement in MRI (25) in diamagnetic CEST or paramagnetic CEST (PARACEST), exchangeable protons resonate at a chemical shift different from that of water. Radiofrequency applied at their frequency saturates exchangeable protons, which transfer into water and reduce MRI signal in their vicinity. Although the sensitivity is rather low, the possibility of switching the signal “on” and “off” has attracted much interest (26).

Magnetic resonance also allows for high sensitivity detection of non-radioactive fluorine (^19^F). Human NK cells were cultured for 24 or 48 h with a commercially available emulsified PFPE perfluorocarbon (CS-ATM-1000) under conditions where labeling had no measurable effect on cell viability and cytotoxicity against K562 leukemia cells. ^19^F-labeled NK cells could then be detected at the site of injection and shown to migrate (27).

### *In vivo* Labeling

Even if *in vitro* cell labeling looks rather easy and if progress has been made, direct labeling of cells prior to injection does not allow for long term *in vivo* imaging. Sensitivity is limited, especially for MRI, when cell viability and functionality is preserved. One drawback has been repeatedly mentioned: macrophages can take up cells or cell debris at the site of injection and migrate. The dilution of the imaging probe during cell division and its release from the cell eventually lead to the disappearance of the signal. Thus, finding alternative routes for tracking cells of interest *in vivo* has been the subject of many technical developments. One such alternative is the *in vitro* cell transfection with genes coding for transporters or enzymes as well as metabolic engineering that allow *in vivo* cell detection using various molecular imaging techniques after injection of a specific tracer.

#### Genetically Engineered Cells for Radioactive, MRI, or Bioluminescence Imaging

To achieve long term labeling, cells can be genetically engineered to express reporter genes. This reporter gene will allow the targeting of the cells by administering an imaging probe. A stable expression of this reporter allows for a virtually unlimited number of imaging sessions, without any impact of cell division.

##### Radioactive imaging

Iodine is taken up by the thyroid and by a few other tissues through the sodium-iodine symporter (NIS). Thus, cells were transfected with the NIS gene, most often the human gene (hNIS), injected and imaged by SPECT using a variety of radioactive tracers including iodine-123 (sodium iodide) and technetium-99m (sodium pertechnetate) in a variety of animal models (28). NIS may also be used for PET with iodine-124 or ^18^F-tetrafluoroborate (29, 30). This approach was used recently to label tumor cells *in vivo* (31) and to monitor dendritic cell traffic from the skin to lymph nodes (32). This approach has some limitations, though. First, as mentioned above, NIS is expressed by a variety of normal cells, particularly in thyroid, salivary glands and stomach. Thus, imaging cells in these organs is excluded due to background signal. Second, sensitivity for the detection and quantification of transfected cells expressing NIS *in vivo* is limited because, in the transfected cells, the radioactive tracer does not become linked to tyrosine as iodine is in the thyroid.

Another reporter gene that has attracted much interest is the herpes simplex virus type 1 thymidine kinase (HSV1-tk). With this kind of genes that code for intracellular proteins, the risk of immune reactions is reduced. HSV1-tk allows for PET and SPECT using a variety of anti-viral agents specific for the virus kinase and not recognized by the human enzyme. They enter cells and become phosphorylated and trapped intracellularly only in HSV1-tk-transfected cells. Compounds such as FIAU (5-iodo-2-fluoro-2-deoxy-1-D-arabino-furanosyl-uracil), FEAU (2-fluoro-2-deoxyarabinofuranosyl-5-ethyluracil) or acycloguanosine derivatives (e.g., FPCV: fluoropenciclovir, FHBG: 9-[4-fluoro-3-(hydroxymethyl) butyl] guanine) may be labeled with ^18^F and used for *in vivo* PET imaging. Sensitivity may be improved by using a mutated gene, HSV1-sr39tk, that codes for a more potent enzyme. HSV1-sr39tk may be used with [^18^F]-FHBG as a tracer (33).

In a similar approach to the transfection of cells with viral thymidine kinase, animals may be engineered to express thymidine kinases in specific cells. As an example, Rosa26-mT/sr39tk mice were generated and HSV1-sr39tk expression in platelets, T lymphocytes or cardiomyocytes was induced. Longitudinal PET imaging and quantification of T-cell homing during inflammation and cardiomyocyte viability after myocardial infarction could then be monitored using [^18^F]-FHBG, a cell-permeable tracer that is phosphorylated by HSV1-tk and retained inside the cells (34).

Alternatively, cells may be transfected to express cell-surface receptors for peptides as, for instance, the human glucagon-like peptide 1 receptor gene and imaged with the peptide labeled with fluorine-18 (35). A similar approach was used to detect transplanted pancreatic islet cells that express glucagon-like peptide 1 receptor (GLP-1R) by PET imaging after the injection of ^64^Cu-DO3A-VS-Cys40-Exendin-4, showing persistent and specific uptake in the mouse pancreas (36). The mutated version of the dopamine receptor, D2R80A, that internalize ^18^F-Fallypride, has also been proposed for imaging mesenchymal stem cells (37, 38).

##### Magnetic resonance imaging

Reporter-gene transfection has been proposed for MRI. The transferrin receptor has been used to capture transferrin-conjugated SPIO particles (39). Dendritic cells transfected with the ferritin gene show increased iron uptake that may be detected by MRI (40, 41). A very similar approach to the NIS system may be used for MRI, by transfecting cells with the Divalent Metal Transporter 1 (DMT1) that can import manganese (42). In the same setting, radioactive manganese (^52^Mn), may be used for PET imaging (43).

##### Optical imaging

Bioluminescence imaging (BLI) consists in the use of a luciferase enzyme, which reacts with its substrate, luciferin, and emits light between 480 and 600 nm, depending on the type of enzyme (firefly, Renilla, or bacterial) and substrate (44). This method implies the insertion of the luciferase gene inside the genome of the tracked cells by cell transfection during *in vitro* culture or by engineering mice to express the luciferase in target cells. In this later case, the mouse itself allow for visualizing intrinsic cells during the development of a pathology. In the case of adoptive cellular therapy, the cells can be isolated from the mouse before the adoptive transfer without need for *in vitro* transfection. Although the insertion and expression of luciferase is stable, so far adoptively transferred cells have only been followed up to a week, due to the decay of the signal. This may be linked to the death of transferred cells (45). However, after the disappearance of the BLI signal, mice were sacrificed, and histology or flow cytometry was performed. It has been reported that, although the cells are still present and express luciferase, the BLI signal is no more detectable (46). Metabolic changes may be suspected as luciferases need energy and cofactors. Due to this lack of sensitivity, BLI is very often associated with another reporter gene, like Green Fluorescent Protein (GFP), which allow the *ex vivo* detection by flow cytometry or immunostaining of the organs.

Indeed, these reporter genes are most of the times not used alone but in association, either to enhance the signal (39) or to confirm its specificity by a different imaging approach (47, 48). Most of these proteins are endogenous and not toxic (dopamine receptor, NIS, ferritin). They can be expressed naturally in some organs of the human body, limiting their use. On the other side, inducing their expression in cells implies a possible impact on the functions of the cells.

Animals may also be made to express fluorescent proteins or luciferase in specific cells. This approach has been extensively developed for many different studies, including oncogenesis and cancer therapy (49). For instance, the photoconvertible fluorescent protein Kikume green-red protein was used to track dendritic cells *in vivo*. The KikGR protein changes its color from green to red upon UV illumination. Then, migration of dendritic cells, specifically CD103^+^ dendritic cells, from the skin to lymph nodes could be monitored after UV illumination of the skin of knock-in mice expressing the protein (50).

#### Metabolically Engineered Cells and Click Chemistry

Metabolic engineering and click chemistry (also known as bio-orthogonal chemistry) takes advantage of fast and high yield chemical reactions that may take place in aqueous media and even *in vivo*. A variety of chemical reagents have been developed that allow for highly specific reactions that are not hindered by biological conditions. Cells of interest were labeled by glycoengineering and bioorthogonal click chemistry by incubation *in vitro* with tetra-acetylated N-azidoacetyl-D-mannosamine to generate unnatural sialic acids with azide groups on their surface. The cells may then be injected *in vivo* and detected by the second click chemistry reagent, coupled to a fluorochrome such as dibenzyl cyclooctyne-conjugated Cy5 (DBCO-Cy5) for near-infrared fluorescence imaging or to iron-loaded nanoparticles for MRI (51). This approach was shown to improve labeling efficacy and to reduce false signals generated by macrophage phagocytosis of *in vitro* labeled cell debris. It does not require genetic modifications. So far, this approach has only been used for near-Infrared fluorescence (NIR) with stem cells and tumor cells (52, 53). Although NIR imaging is non-toxic and cheap, its limited spatial resolution and poor penetration through tissue complicate its use in clinical imaging.

#### Indirect Methods: Labeled Antibodies and Tracers

Labeled antibodies may be used to detect cells *in vivo* by SPECT, PET, or NIR fluorescence. They have mainly been used for tumor diagnosis, staging or tumor response monitoring (54). It has been reported that labeled antibodies allow the tracking of T cells *in vivo* (55).

The first step is to choose the target antigen. Ideally, this antigen should be exclusively expressed on target cells, but most of the time other tissue also express it. For T lymphocytes, many targets have been tested, e.g., CD3, CD8, CD2, and CD7 (56–58).

Once the target is chosen, the antibody must be radiolabeled. Ideally, the radionuclide has a half-life compatible with the biological half-life of the antibody. In human, ^89^Zr and ^64^Cu, with half-lives of 78.4 and 12.7 h, respectively, have been used for PET imaging. The radiolabeling method also has an important impact on the quality of the images, since free radionuclide can lead to enhanced background noise, or worse, false positive signal in normal organs, where the target antigen is not expressed. For instance, ^89^Zr shows a natural tropism to the bone (59) that can impede the tracking of bone marrow cells.

Multistep labeling techniques using antibodies have been developed to improve target to normal tissues ratio. Among these pretargeting approaches, the affinity enhancement system (AES) has been shown to be an excellent method for *in vivo* tumor imaging by SPECT and PET (60). Recently, new pretargeting approaches have been developed. One is based on the *in vivo* formation of an oligonucleotide duplex. A first oligonucleotide analog (e.g., peptide nucleic acid or PNA) is coupled to an antibody or a small binding protein (e.g., an anti-HER2 Affibody) for pretargeting of a radiolabeled complementary oligonucleotide analog (61). Another approach is based on bio-orthogonal chemistry (62). The CC49 antibody recognizing the tag72 antigen derivatized with trans-cyclooctene (TCO) was used for pretargeting ^111^In-labeled DOTA-dipyridyltetrazine, demonstrating fast and high tumor activity uptake and high tumor to muscle ratio in a mouse model. Using small binding proteins such as diabodies or affibodies instead of intact IgG antibodies improves the pretargeting performances for PET (62, 63). Pretargeting may also be applied to NIR fluorescence imaging (63).

The feasibility of detecting cells *in vivo* using MRI and contrast agents targeted using antibodies or antibody fragments has been tested. Magnetic iron oxide nanoparticles were coated with ethylene oxide polymers and coupled to a ScFv targeting the epidermal growth factor receptor. The product showed a long blood circulation time and low accumulation in liver and spleen. Although *in vitro* binding and internalization was specific, 24 h after administration to mice bearing EGFR-positive breast cancer 4T1 mouse mammary tumors, MRI signal reduction resulting from uptake of the reagents in the tumor was observed but this signal reduction was equivalent for the targeted and the control products (64). More recently, the same approach was improved by site-selective scFv conjugation to SPION PEG nanoparticles. *In vivo*, the decrease of MR signals in HER2^+^ xenograft tumor was about 30% at 24 h after the injection, while non-targeted SPION PEG nanoparticles showed no effect (65).

### Bi(multi)Modal Imaging

Multimodality approaches deserve specific attention, even if they are generally limited to preclinical studies. Not only can they combine various imaging modalities, such as radioactive, MRI or optical imaging, but also *ex vivo* and *in vivo* labeling as well as post-mortem studies. Thus, bimodal systems have emerged that combine magnetic resonance imaging (MRI) or PET with fluorescence or bioluminescence.

Genetically engineered dendritic cells (DC) have been developed for MRI. Proteins which have an affinity for iron compounds may be used as MRI reporters. In a recent study, DC were engineered to express human ferritin heavy chain (FTH), which chelates iron and acts as an endogenous MRI contrast agent, and GFP genes to allow both fluorescence and MRI cell tracking (40). Reporter genes can also be an enzyme like the Drosophila melanogaster 2′-deoxynucleoside kinase (Dm–dNK) that phosphorylates native deoxynucleosides and a wide range of synthetic nucleoside analogs, including fluorescent nucleosides (66). In this study, the fluorescent nucleoside analog, 2′-deoxycytidine (pyrrolo-dC), generated highly specific CEST MRI signal and fluorescence for bimodal imaging (67).

DC can be loaded by phagocytosis of an antigen labeled with an MRI contrast agent (68). It is possible to effectively load DC with multifunctional polymeric nanoparticles. Nanoparticles composed of iron oxide bearing the OVA antigen coupled to a NIR fluorophore (MNP-OVA) allowed the monitoring of the migration of DCs to lymph nodes in DC adoptive transfer immunotherapy using NIR fluorescence imaging and MRI (69).

PET tracking of genetically engineered DC in combination with bioluminescence has also been developed. In a study, DC were made to express both human NIS and effluc genes. DC migration is then made possible by using ^18^F-tetrafluoroborate (TFB), a substrate for the NIS reporter gene. Bioluminescence imaging is performed to confirm PET results (32). A combination of PET and Cerenkov luminescence has also been described (70).

Non-phagocytic regulatory T cells (Tregs) have been imaged *in vivo* after transduction by human NIS and the fluorescent protein mCherry. NIS expressing Tregs were labeled *in vitro* with technetium-99m pertechnetate (^99m^TcO4^−^) and imaged *in vivo* in C57BL/6 mice by SPECT/CT. After 24 h, Tregs were detected in the spleen and the bimodal labeling confirmed their localization by organ biodistribution studies and flow cytometry (71). In a similar way, bone marrow stem cells were labeled with gadodiamide (Omniscan), a non-ionic complex of gadolinium, using the fluorescent Arrest-In transfection reagent (72).

Nanoparticle systems can integrate therapeutic and imaging agents in a single formulation. They may be particularly useful as multimodal imaging agents. They have been used to deliver these agents through passive or active targeting to cells *in vitro* and *in vivo*. The different kinds of such nanoparticles, which include polymeric nanoparticles, micelles, liposomes and dendrimers and their potential applications in cancer immunotherapy, and immune cell tracking have been reviewed in detail (73).

## Cell Tracking Achievements: What Happened in Cell Tracking Over The Last Ten Years?

New methods have been developed, but has *in vivo* cell tracking advanced (cancer) immunotherapy? *In vivo* imaging has the potential to contribute as a drug development tool to improve the understanding of complex mechanisms of action, as a tool to improve efficacy, for example, by stratifying patients as possible responders or non-responders, and as a non-invasive treatment response biomarker to guide immunotherapy and recognize early signs of loss of efficacy. In cell therapy, a series of questions are asked about the delivery of the cells, their viability, differentiation of proliferation, as well as about the immune responses they may trigger. At this point, preclinical studies have been numerous, but transfer to the clinic remains quite limited (74). This part of the review aims at providing a non-exhaustive survey of achievements in cell tracking using the current tracking methods summarized in Table 1.

**Table 1:** Current tracking cell methods in pre-clinic, depending on the type of labeling (direct, indirect, and transgene) and the modality of imaging (TEP/SPECT, MRI, BLI/fluorescence, and multimodal imaging).

	**Direct labeling**			**Indirect labeling**				**Transgene**			
	**Radioactive/contrast agent**	**Cell type**	**References**	**Radioactive/contrast agent**	**Targeting molecule**	**Cell type**	**References**	**Transgene**	**Radioactive/contrast agent**	**Cell type**	**References**
TEP/SPECT	Zirconium 89 Indium-111 Cuivre-64 18-FDG 18F-FLT Rie-AuNPs	Dendritic cells T cell Leukocytes Glioma cells Cardiac stem cells HUVECs Macrophages	(10) (10, 14–16, 75) (11) (12) (17) (18) (76)	Copper-64 and Zirconium-89 lnidum-111 Lutetium-177 Carbon-11	Radiolabeled antibody anti CDS, CD7, PDl, CD3 anti HER2 affibody rituximab anti TAG72 diabody Diolabeled antibody anti F4	Tumor-infiltrated lymphocytes Tumor cells Macrophages	(56, 77–79) (61) (62) (63) (80)	Transporter NIS GLP-lR D2R80A HSVl-tk	18F -tetra fl uorobora te Copper-641abeled peptide 18F-Fallypride Fluor-18	Dendritic cells and tumor cells Pancreatic cells Stem cells T cells and cardiomyocytes	(31, 32) (36) (37, 38) (34)
MRI	SPIO Gadolinium-DTPA MPIO, USPIO Fluorine-19	Dendritic cells Macrophages T cells MSC Macrophages NK cells	(22) (81) (24) (20) (23, 76, 82) (27)	Iron nanoparticles	Click chemistry	Stem cells	(53)	Transferrin receptor Ferritin	SPIO particles Iron uptake	Human Mesenchymal Stem Cells Dendritic cells	(39) (40, 41)
BLI/fluorescence				ManNaz and DBCO	Click chemistry	Stem cells Tumor cells	(52, 53) (63)	Luciferase Kikume green-red		Stem cells T cells Macrophage Dendritic cells	(34, 46) (83) (84, 85) (50)
Multimodal	Fluorescence+ MRI	Dendritics cells :*ex vivo* labeling tumor peptide	(69)					Fluorescence+ MRI: DMdNk protein PET+BLI:NIS TEP/IRM: transporteur DMTl PET+ fluorescence: NIS and mcherry gene	CEST MRI 18 tetrafluoroborate Manganese-52 99mTC04-	Genetically engineered DC Human stem cells Regulatory T cells	(66, 67) (32, 70) (43) (71)

### Investigating the Tumor and Its Microenvironment

The evaluation of tumor volume and demonstration of tumor shrinkage remains the basis for tumor response assessment with the so-called RECIST criteria. It can be easily performed by CT-scans or MRI when the lesions are measurable, which is by far not always the case. In addition, tumor shrinkage may be delayed and some effective treatments (e.g., some kinase inhibitors) do not result in prominent tumor volume changes. Alternative response criteria, PERCIST, have been proposed (86). In addition, new imaging technologies offer possibilities to look at tumor lesions not as a non-descript mass of tumor cells, but as a complex body of interacting cells of different origins.

#### Imaging Tumor Cellular Composition

Measuring the relative number of tumor cells in the tumor lesion before and after treatment, may be useful in response assessment. Highly specific markers are needed. For instance, compounds that target melanin biosynthesis (benzamides) (87) and metallopeptides (88) binding to melanocortin type 1 receptor (labeled MSH analogs) have been used in melanoma, but many other labeled molecules, including antibodies, labeled for SPECT and PET, have shown high imaging performances in terms of sensitivity and specificity (89, 90).

#### Imaging TILs

Monitoring the phenotype and function of tumor infiltrating lymphocytes has long been recognized to be important in adoptive tumor cell therapy (91). This was achieved, in animals as well as in human, by the administration of radiolabeled tracers, usually antibodies or analogs, and SPECT or PET. For example, ^64^Cu-labeled diabody specific for CD8 was used to assess CD8 T cell density in tumors in mice and treatment related changes (92). Whole antibodies, labeled with zirconium-89 afford similar results (56). Many target antigens have been tested in animal models (56, 58) and CD7 seems so far to be the best candidate to target T lymphocytes, with the lowest toxicity (56).

Surprisingly, in patients, immuno-PET has not been used to detect lymphocytes in tumors, other than through their expression of immune checkpoints, as discussed below. However, labeled IL-2 has been used to visualize lymphocyte infiltrating tumors (77, 93). In a pilot study, patients with metastatic melanoma receiving ipilimumab (IPI) or pembrolizumab (PEMBRO) were subjected to SPECT/CT imaging with ^99m^Tc-labeled interleukin-2 in an attempt to detect TILs. In 5 patients (2 treated with IPI and 3 with PEMBRO), metastatic lesions could be visualized with a positive correlation between size and ^99m^Tc-HYNIC-IL2 uptake, both before and after 12 weeks of therapy (93).

Texture analysis and radiomics may also, without administration of tracer, provide molecular information about infiltration of lymphocytes in tumors. In cancer patients, evidence of for the presence of CD3 T cells in tumors have been obtained by MRI texture analysis (94) and for the presence of CD8 T cells by CT radiomics and Artificial Intelligence analysis (95).

#### Macrophages

Macrophages are tissue-resident cells of the innate immune systems that perform a variety of functions in host tissue repair and maintenance of homeostasis. Macrophages are associated with auto-immune and inflammatory diseases and, in oncology, one of the tumor escape factors is the presence of pro-tumor macrophage, tumor-associated macrophages (TAM) that support tumor growth (96). *In vivo* studies have analyzed the biological role and migration of macrophages using different imaging methods such as fluorescent imaging (97), PET, MRI, and multimodal imaging. Macrophages migration to the inflammatory site after an induction of inflammation was analyzed by *in vitro* labeling with radioactive iodide-embedded gold nanoparticles (RIe-AuNPs) and PET imaging (76). During inflammatory disease such as arthritis, atherosclerotic plaques, *in vivo* staining of the macrophage with ^111^In- or ^64^Cu-labeled antibodies allowed imaging follow-up, evaluation of therapeutic efficacy and therapy adaption (98, 99). For acute or chronic obstructive pulmonary disease, the recruitment of macrophages was monitored by labeling with amine-modified PEGylated dextran-coated SPIO and MRI (81).

In oncology, macrophages are an important part of the tumor microenvironment and thus a therapeutic target. Indeed, the presence of TAM favors tumor escape. In order to assess their presence in tumors and to analyze the efficacy of therapy, these cells were tracked by immuno-SPECT using ^111^In-labeled anti F4/80 (100) antibody, by MRI using the contrast agents MPIO (82) and ultra-small iron oxide nanoparticles (USPIO) (101), by BLI in transgenic luciferase mice (84) or by multimodal imaging combining MRI and BLI (85). PET imaging using labeled ligands targeting receptors overexpressed in macrophages, such as the Translocator protein (peripheral benzodiazepine receptor), has also been proposed (80).

#### Imaging Tumor Metabolic Activity

^18^F-FDG is the most commonly used radiopharmaceutical for imaging tumor metabolism in clinical practice. Its use is based on the increased glycolytic rate in tumors compared to physiologic cells, known as the Warburg effect. However, inflammatory and other metabolically active effector immune cells may contribute to activity uptake in tumor lesions (102). By contrast, lesions with high numbers of proliferative tumor cells are ^18^F-FDG avid, whereas low ^18^F-FDG avid lesions have been shown by immunohistology to be infiltrated by activated immune cells. As a result, ^18^F-FDG is not considered as a marker of immune response and new markers such as amino acids, nucleotides, choline, and receptor ligands have been studied. In hematolymphoid tissues, however, increased levels of deoxycytidine kinase (DCK) expression is found; DCK is the rate-limiting step in the deoxycytidine salvage pathway. The tissue-specific expression of this enzyme allows more specific targeting by, for example, ^18^F−2-fluoro-D-(arabinofuranosyl)cytosine (^18^F-FAC), which has been shown to accumulate preferentially in CD8^+^ T cells and in innate immune cells in mice (103).

^18^F-labeled 3-fluoro-3-deoxythymidine (^18^F-FLT) is trapped intracellularly after phosphorylation by thymidine kinase 1 (TK-1) but is not incorporated into DNA since ^18^F-FLT-monophosphate is a very poor substrate of thymidylate kinase (TMPK). Imaging with ^18^F-FLT has been evaluated to show proliferation more specifically (18), but effector immune cells that infiltrate tumors are mostly of a differentiated phenotype and do not proliferate. Thus, ^18^F-FLT uptake in the lymph nodes of vaccinated patients only increased in the presence of antigen-loaded DC, providing the first clinical demonstration that immune responses induced by antigen-specific therapy can be imaged *in vivo* (102).

In bladder cancer, and presumably in other cancers, correlations have been observed between tumor ^18^F-FDG uptake and expression of PD-1/PD-L1 (104). Such a correlation may be useful for the selection of appropriate therapeutic strategies.

### Immune Checkpoint Inhibitors: Assessment of Immune Status in Tumor Lesions

Immunotherapy agents do not directly attack tumors but re-activate the immune system by targeting adaptive or innate immunity. Immuno-oncology has been revolutionized by the introduction of immune checkpoint inhibitors (ICI) and the approval of ipilimumab in 2011. ICI are monoclonal antibodies targeting immuno-regulatory molecules on the surface of T cells, antigen-presenting cells, and neoplastic cell populations. Clinical success of reagents blocking the CTLA-4 (cytotoxic T lymphocyte-associated protein 4, CD152) and PD-1/PD-L1 checkpoints (programmed cell death protein 1, CD279; programmed death-ligand 1, CD274) has driven rapid regulatory approval for treatment of patients with both solid and hematologic malignancies (105). Patients treated with immune checkpoint inhibitors (ICI) have objective response rates of 20–40% for solid tumors, lymphomas, and malignant melanomas. Thus, 60% of patients do not respond to treatment. It may of course be expected that patients with tumors presenting a higher load of tumor infiltrating lymphocytes (TIL) are more likely to respond to anti-PD-1/PD-L1 check point inhibitors (106).

A detailed understanding of the tumor microenvironment, including the identification and quantification of different immune cell subsets, their spatial context, and the expression of these immune checkpoint markers is obviously required to go further with these new therapies (107). Changes in immune cell infiltration and biomarker expression before and after therapeutic intervention are critical parameters for clinical development (108). Thus, assessment of PD-L1 expression by IHC has emerged as an important predictive biomarker for patients with various cancers including non-small cell lung cancer (NSCLC) and renal cell cancer (78).

Immuno-detection using antibodies labeled with zirconium-89 or copper-64 for PET, as well as indium-111 for SPECT, has been used to assess the CTLA-4 and PD-1 status of TIL *in vivo* and the expression of PD-L1 by tumor cells in order to predict the therapeutic efficacy of the administration of immune checkpoint inhibitors in mice and in human (79, 109–111). This approach was also proposed in the context of anti CTLA-4 therapy (107). Based on tumor biopsies, it appears that some patients with PD-L1-negative tumors show clinical benefit of anti-PD-L1 treatment. Thus, a zirconium-89-labeled anti-PD-L1 antibody (atezolizumab), was used to image 22 cancer patients before atezolizumab therapy. High PET signal was observed in lymphoid tissues and inflammation sites. In tumors, high but heterogeneous and variable across tumors uptake was observed and clinical responses could be better correlated with PET than with immunohistochemistry or other biomarkers (112).

In mice, the presence of CD8+ T-cells was monitored using ^89^Zr-labeled an anti-CD8 single domain antibody after treatment of B16 melanoma with an anti-CTLA-4, showing that response correlated with the homogeneity of the distribution of CD8+ T-cells through the tumors (58). In mice with B6-F10 syngeneic melanoma, an anti-mouse PD-1 antibody labeled with copper-64 showed tumor uptake (79).

### Monitoring the Activation and Expansion of Immune Effector Cells

Activation and expansion of the immune system may be monitored by imaging changes in the expression of various receptors to cytokines and growth factors as well as changes in the amounts of interstitial water resulting from inflammation. Immune cell trafficking is another aspect of immune system activation.

#### Imaging Immune Cell Activation

Several examples may be found in preclinical and clinical studies. In mice, an antibody against the cytokine IFNγ, which becomes sequestered at the surface of tumor cells after its production by T lymphocytes, was shown to reflect the activation status of cytotoxic T cells (113).

Reactive lymph nodes also express and secrete chemokines that induce immune cells relocation. Among others, the CCR7 chemokine and chemotactic agents, which play a key role in directing cell trafficking, are suitable imaging targets. For example, CXCL12 is a key chemotaxis factor for lymphocytes with expression of the CXCR4 receptor on their cell membrane (114). PET tracers targeting CXCR4 were thus used in cardiovascular disease and infections. Interestingly, Radiolabeled CXCR4 ligands are also very effective for cancer cell imaging (e.g., ^68^Ga-labeled pentixafor) and CXCR4-trageting therapeutics labeled with ^177^Lu are currently under clinical development (114).

Activation of the immune system also results in VEGF release and, subsequently, in significant lymph node volume increase. Lymph node volume can be measured using various techniques including MRI, CT, and ultrasound. Ultrasound imaging using targeted microbubbles improves the evaluation of the microvasculature (115). Dynamic contrast-enhanced (DCE)-MRI using gadolinium (Gd) or USPIO-based contrast agents may also be used to monitor angiogenesis: expansion of lymph node size, total blood flow and blood volume, permeability of perfused capillaries, and total surface of perfused capillaries. MRI measures of vascularity using iron-based contrast agents have been validated against histology, the gold standard in angiogenesis assessment. Diffusion-weighted (DW)-MRI detects metastatic lymph nodes (116) and may be able to image reactive LNs in immune responses.

Imaging the expression of VEGF receptor may also be a way to monitor the activation of endothelial cells in LN resulting from immunotherapy. This was achieved in preclinical models by using anti-VEGFR (bevacizumab) labeled with indium-111 for SPECT (117) or by using RGD peptides labeled with various radionuclides for SPECT and PET imaging (118, 119). These approaches have shown potential in mice, for instance, to image inflammation-induced expansion and regression of lymphatic networks by PET, they have not yet been translated into human.

Changes that occur in the tumor due to an increased immune response can also be imaged using MRI, for example through changes in relaxation times, contrast, or apparent diffusion coefficient (120). These changes have been shown to correlate with conventional histological measures in mice after treatment by transferred cytotoxic T cells that expressed a modified TCR specific for a tumor antigen.

#### Imaging Trafficking of Immune Effector Cells

Antigen presenting cells (APC) are cells of the immune system that present pathogen peptides linked to class I or class II major histocompatibility complex (MHC) molecules to T lymphocytes (TL) to initiate adaptive immune responses. They are dendritic cells (DC), macrophages, and B lymphocytes. Analyzing antigen capture, migration to the lymph nodes and antigen presentation by APC started with fluorescently labeled cells using *in vivo* intravital optical imaging (121, 122). Regardless of the microscope type used, this system remains an invasive process, limited in depth penetration and restricted to a specific area of the body. Thus, APC trafficking has been monitored mostly by MRI and, more recently, tracking methods using PET have been reported. Either the cells or mice are genetically engineered, or labeled antigens are loaded *in vivo* or *ex vivo* into APC thanks to their phagocytic capacity. DC may be loaded *ex vivo* with pathogen peptides or irradiated tumor cells and reinjected to the patient. As an alternative, vaccination using labeled irradiated tumor cells or inactivated pathogens have been used to quantify antigen capture and delivery to lymph nodes by MRI (123).

Imaging lymphocyte trafficking is most easily achieved with *ex vivo* labeled cells. Transfused cells often traffic initially to the lungs, bone marrow, liver, and spleen. In mice, labeling Th1 cells with ^64^Cu-PTSM was shown to permit their detection in single LNs and to monitor T-cell homing *in vivo* over 48 h (117). Changes in cell trafficking resulting from treatments with cyclophosphamide or IL-12 may be monitored by *in vivo* imaging. A similar method, using zirconium-89, was used to monitor γδ T-cells homing into tumor lesions in mice (75). IL13Rα2-CAR T cells delivered intraventricularly were detectable by PET for at least 6 days throughout the central nervous system and within intracranial tumors. When intravenously administered, PSCA-CAR T cells also showed tumor tropism, with a nine-fold greater tumor-to-muscle ratio than for CAR-negative T cells. Bone marrow uptake of ^89^Zr-labeled hematopoietic stem cells could also be monitored in mice (124) and bone marrow cell uptake in acute fractures in mice could be inhibited, rather than accelerated, by a CXCR4 antagonist, plerixafor (125).

The use of reporter gene expression is another way to study cell trafficking, because imaging is independent of factors lifetime and distribution of the tracer and an enzymatic reporter allows for amplification of a weak signal. Antigen-specific T-cells were made to express a viral Tk gene could be tracked in mice, over a period of 3 weeks, using an ^18^F-tagged probe specific to this variant of Tk. Detection of 10^4^ T cells was claimed (67).

Lymphocytes may also be imaged by targeting cell surface markers. ^99m^Tc-labeled IL-2 was used to detect tumor-infiltrating lymphocytes in melanoma patients (77, 93). Non-depleting ^111^In-labeled anti-CD4 antibodies have been used to track CD4^+^ T cells by SPECT in mice with good correlation with pathologic measures (126). *In vivo* ^19^F MRI was also used to track homing to draining lymph nodes of T cells that were intracellularly labeled *ex vivo* with a perfluoropolyether (PFPE) nanoemulsion (127). Time-lapse ^19^F MRI was used to calculate the number of T-cells in lymph nodes over 21 days and correlated with *in vitro* fluorescence measurements to compensate for *in vivo* T-cell division. MRI also allowed visualization of CD8^+^ cytotoxic T cells, regulatory T cells, and myeloid-derived suppressor cells loaded with to monitor the effect of vaccination. Increased recruitment of cytotoxic T cells and decreased recruitment of myeloid-derived suppressor cells and regulatory T cells to the tumor was observed (128).

### Cell-Based Therapies

#### Earlier Results in Cell-Based Therapy

So far, most clinical studies have used ^99m^Tc or ^111^In or superparamagnetic iron oxide to label therapeutic cells for *in vivo* cell tracking using SPECT or MRI, as reviewed by Srinivas et al. (129). Adoptive T-cell therapy (ACT) using expanded autologous tumor-infiltrating lymphocytes (TIL) and tumor antigen-specific T cell expanded from peripheral blood are complex but powerful immunotherapies. Clinical trials that included cell tracking have compared various routes of administration, the effect of the number of injected cells or host pretreatment with cyclofosfamide and compared various therapeutic cell preparation and encapsulation methods.

#### Tracking Antigen-Presenting Cells *in vivo*

DC are the most effective professional antigen presenting cells for the priming of naïve T cells *in vitro* and *in vivo*. These properties are the consequence of constitutive expression of MHC molecules class I and II and co-stimulatory molecules (CD80, CD86, CD40) and of their ability to secrete regulatory cytokines such as interleukin 12 upon recognition by the T cell receptor. In the immature stage, DC have the ability to capture the antigen by phagocytosis or endocytosis, migrate to the lymph nodes where they become mature and prime T lymphocytes inducing the adaptive immunity. This is the reason why, in recent years, immunotherapy targeting dendritic cells has developed.

Imaging demonstrated the ability of intradermally or subcutaneously administered therapeutic DC to migrate from the sites of injection into lymph nodes with about 4% of DC reaching draining lymph nodes (130). Actual contact of the DC with T-cells cannot be demonstrated by *in vivo* imaging, but *ex vivo* only after lymph node resection. By contrast, intravenously injected mature DC are trapped in the lungs and redistribute to the liver, spleen, and bone marrow. No lymph node localization has been detected so far, which does not mean that DC completely fail to reach the lymph nodes. The techniques may not be sufficiently sensitive to detect the small numbers of cells that do reach the lymph nodes. Direct intranodal administration of therapeutic DC is also common in clinical studies. Then *in vivo* imaging has been used to study the migration of DC from the primary injected node to secondary nodes. The large variability in the fraction of injected cells (from 0 to 84 %) that was shown to migrate cast doubts on the accuracy of intranodal injections. Labeling the antigen to monitor its fate after DC delivery has been proposed in preclinical *in vivo* models. When DTPA was conjugated to the epsilon NH_2_ group of the Lys154 residue, MHC binding of the peptide was preserved and could still be recognized by cytotoxic T cells. These studies allowed the non-invasive determination of the behavior of MHC–peptide complexes expressed by DC in cell vaccination (131) but has not yet been reproduced in the clinic.

#### CAR (Chimeric Antigen Receptor) T-Cell Therapy

CAR T-cell therapy is a fast-growing research field with various therapeutic indications in autoimmunity, allotransplantation, infection and cancer. Enhancing the functionality and the safety of the injected cells is an important aspect of the clinical development of this very potent therapy. Therefore, there is a real need to develop *in vivo* molecular imaging to better visualize, predict and improve the efficiency of this type of immunotherapy (5). However, so far, clinical studies of CAR T-cell tracking have only established proofs of concept of its feasibility (74).

SPECT and PET imaging are two possible modalities for tracking the fate of T-cells injected for therapeutic use. Labeling T-cells has been extensively investigated and radiolabeling is possible with little impact on cell function or migration ability (13). However, the radionuclide half-life is a limitation to track the cells more than for a few days after injection.

A variety of solutions to this limitation have been proposed (132). While multimodal imaging has been shown possible (133), CAR T-cell tracking in animals has demonstrated homing and persistence in the tumors and spleen by *ex vivo* MRI of tissue samples after CAR T-cell labeling with perfluorocarbon (134) and in whole animals by immuno-PET (135). Although the context of CAR T-cell therapy would be appropriate to develop genetic modification of the T-cells to express reporter genes as discussed above, the outcome of therapy remains monitored mostly by functional imaging and especially by MRI (136).

#### Other Adoptive T-Cell Transfer Therapies

Although BLI is most of the time used to follow tumor and stem cells, T cells could also be monitored thanks to the luciferase gene; for instance, the migration of CD8 T cells toward tumor site was evaluated in a xenograft mouse model (83), and the migration of tumor associated macrophages has been visualized in a transgenic mouse model of ovarian cancer (84). Also, by optical imaging, but using a fluorophore targeted to NIS-transfected cells, tracking of *ex vivo*-expanded NK cells has been performed *in vitro* and *in vivo* showing fast NK cell accumulation in tumors in triple-negative in breast cancer xenografts (137).

^89^Zr-oxinate labeling was used to track V_γ9_V_δ2_ T cells *in vivo* by PET. In a mouse xenograft model of human breast cancer, the V_γ9_V_δ2_ T cells could be tracked over 1 week and it was shown that injection of PEGylated liposomal alendronate increased homing of the T cells to the tumors, which was confirmed by histology (75).

#### Stem Cell Therapies

Mesenchymal stem cells (MSC) have been proposed for cardiac regeneration after myocardial infarction (MI). Mesenchymal stem cells derived from rat fetal heart have the potential to differentiate into cardiomyocytes, endothelial cells and smooth muscle cells *in vitro*. These cells were labeled with technetium-99m for *in vivo* tracking that revealed a focal uptake of cells in the anterior mid-ventricular region of the heart in line with subsequent ventricular functional recovery (138). Cardiac stem cells were also loaded with ^18^F-FDG and imaged by PET to quantify their biodistribution and assess the retention of implanted cells in a model of chronic myocardial infarction in pigs. Acute cell retention was shown not to correlate with cell engraftment, which is improved by IM injection (17).

Stem cells have been tracked in various models with BLI, for instance in acute liver injury or acute kidney injury, to study the migration and persistence of human bone-marrow derived stem cells to the liver and kidney (45, 139). Luciferase-transfected adipose-derived stem cells could be transplanted in liver and brain and monitored *in vivo* by bioluminescence for several days. *Ex vivo*, immunofluorescence detected the continued expression of luciferase for 4 months, demonstrating that the transplanted cells do not dye, even if the bioluminescence signal is lost (46).

In a tumor graft model, the migration of mesenchymal stem cells toward the subcutaneous tumor could also be observed (140). This study took advantage of a different type of luciferase that metabolizes different substrate, allowing them to follow the migration of stem cells with the Firefly Luciferase, and the tumor progression with the Renilla Luciferase.

BLI imaging has allowed to investigate the impact of stem cells injection modalities, showing that the intravenous route often leads to sequestration in the lung (141) preventing the migration of stem cells to other organs, while the intracardiac route seems to prevent this phenomenon.

## Discussion

Cell tracking has a long history of routine clinical use in Nuclear Medicine and it serves a purpose in infectious and inflammatory diseases despite its limitations (142). Imaging has an increasing role in the context of personalized medicine, which becomes the approach to take, at least in developed countries. CT-scan, MRI and ultrasonography are now inescapable and Nuclear Medicine modalities have gained larger recognition, particularly in cancer. However, the number of tracers of frequent, routine use remains quite limited. In addition to bone and thyroid scans, ^18^F-FDG is certainly the tracer that has the biggest impact in cancer management, with a few other PET tracers for those cancers in which ^18^F-FDG does not perform so well, such as prostate cancer. In view of the incredible number of preclinical and early clinical studies about cancer imaging, this seems not much. There are many obvious reasons, the major one being the difficulty of demonstrating that a new imaging technique has its place in medicine as compared to all existing ones. If the imaging technique needs an injectable tracer, such as a radiopharmaceutical, the situation is worse, because of the cost of developing a product that has the regulatory status of a drug and by far not the sales and price of a therapeutic compound.

Will immunotherapy change this situation? Most of the very large number of original publications and reviews that deal with immunotherapy advocate for more imaging, especially more specific imaging of receptors, antigens and other biomarkers that characterize the function of cells *in vivo*. With the progress of cellular therapies, whether regenerative or cancer-oriented, many papers call for cell tracking *in vivo* as a way to understand their behavior and mechanisms of action and, by the way, to design improved therapies. Indeed, if not all novel immunotherapies are cell therapies, they all bear upon complex cellular interactions at the tumor sites and in immunologic tissues and better knowledge of the nature of cancer and non-cancer cells residing in tumors, their activation, proliferation and migration in living animals and, of course, in humans, must be a way of progress in therapy.

This realization triggered a lot of developments that made possible better cell labeling, mostly to make them visible by MRI and PET, for longer times after re-injection, as well as improved tracers to target specific biomarkers *in vivo*, using SPECT and PET, but also MRI, not only on tumor cells but on those cells that make the tumor microenvironment, e.g., endothelial cells, infiltrating antigen-presenting cells, lymphocytes, macrophages and other cells of the immune system. New techniques have been developed and the use of reporter genes to make cells detectable any time after their inoculation using specific tracers is a particularly elegant and powerful one. This review has rapidly depicted these approaches and it is expected that it convinces the reader that they are feasible and effective.

There is no best technique, though. It depends on the objective and, obviously, the most powerful one, for instance the use of reporter genes, are associated with complex manipulations, cost and regulatory hurdles. Interestingly, radioactive (SPECT, PET) and non-radioactive (MRI, optical imaging, ultrasonography) methods have been proposed, which all have advantages and drawbacks. Of course, bimodal and even multimodal agents have been developed. Multimodal imaging is clearly the way to go, with SPECT and PET now always associated with CT and PET-MRI systems developing. It is also clear that multimodal imaging experiments in animals that allowed for *in vivo* imaging and *ex vivo* in-depth investigation of the fate of injected cells and confirmation of *in vivo* imaging results are most convincing. However, such studies do not necessarily need bifunctional tracers, which sometimes look more like “tours de force” than candidates for further development.

One question here is: have these developments and studies been useful for the development of immunotherapy? The answer is not obvious. Such studies have pointed out to some problems and they have been mostly confirmatory, when they have not merely been proofs of concept for feasibility. This review has attempted at presenting clinical results in cell tracking and, while it may have missed some, it is in line with other recent reviews on the subject to conclude that the number of clinical studies is quite small. It may be considered that this is only the beginning of a new story and that groundbreaking discoveries in immunotherapy will be made thanks to imaging and that at least some of the approaches reported here will find their application. Very few cell tracking techniques will become routine. The introduction of reporter genes in therapeutic cells is probably the technique with the highest sensitivity for long term monitoring of cell trafficking, proliferation, and persistence. For cell therapies in which the cells are genetically modified, namely CAR-T cells, the addition of a second gene and cell tracking may be considered in the context of clinical trials. Whether this approach will be used in routine clinical practice is not likely. Conversely, tracers for *in vivo* imaging, particularly PET imaging, designed to detect and quantify specific cell populations are being developed and some will find a routine use. It is always difficult to make predictions, but it seems logical that expensive therapies or therapies that may be efficacious but associated with serious side-effects will not be given to patients who have no chance to benefit from them. The imaging of tumor microenvironment may give answers to how the patients will respond to such therapies, especially immunotherapy. Expression of immune checkpoints, like anti-PD-1, is already assessed from biopsies prior to immunotherapy, but the use of PET-imaging or MRI could allow a non-invasive assessment of the immune state of the tumor. This could provide new insights into the prediction of the response to treatment in patients. This is the theragnostic approach, which is not a reality today, but most probably be one in the future. It is also quite probable that future research in immunotherapy will take advantage of all these technological advances, certainly for preclinical studies, but also in the clinic. Indeed, it is time to combine the novel therapeutic approaches, which afford impressive remissions but not yet to all patients and this will call for precise, specific understanding of what is really going on in the living organism.

## Author Contributions

All authors listed have made a substantial, direct and intellectual contribution to the work, and approved it for publication.

### Conflict of Interest

The authors declare that the research was conducted in the absence of any commercial or financial relationships that could be construed as a potential conflict of interest.
